# Supplementary figures and images for: Hypoxia-inducible C-to-U coding RNA editing downregulates SDHB in monocytes
Source: PeerJ. 2013 Sep 10;1:e152. doi: 10.7717/peerj.152 (PMC3775634; doi:10.7717/peerj.152)

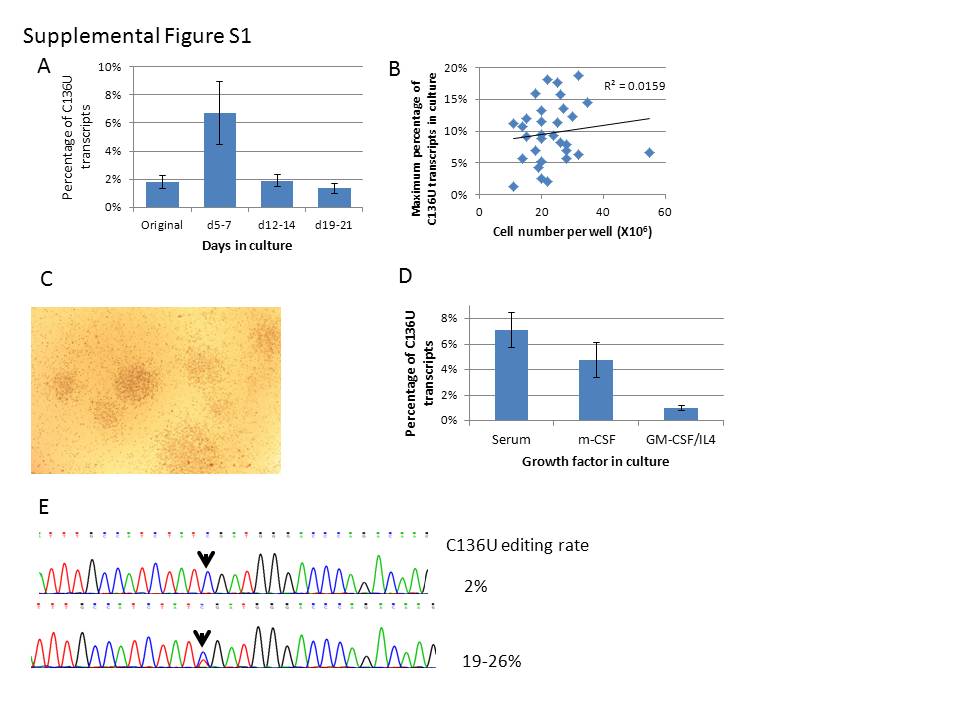

Supplement: Figure S1 — (A) Impact of long-term culture and cell density on C136U editing are shown. Five monocyte-enriched PBMCs were cultured long-term and the C136U editing rates were measured. The editing rate was higher in adherent cells on days 5–7 than in the uncultured monocyte-enriched PBMCs and on days 12–14 and 19–21 (P = 0.017, n = 5, one-way ANOVA). (B) Maximum C136U editing rate in the adherent cells shows very weakly positive correlation with the total number of monocyte-enriched PBMCs per well. Total number of samples = 31. (C) Attached aggregates (AAs) on culture day 20 are shown. The AAs start loosening after one week in culture and eventually appear as flat areas of increased cellular density. This culture was fed with fresh RPMI-1640/10% FBS on day 8. (D) Cytokine mediated differentiation towards macrophages or dendritic cells reduce C136U editing rates. Cultures containing 10% FCS showed higher mutation rates than those treated with M-CSF (p = 0.012) or GM-CSF/IL4 (p = 0.04). Multi-day averages from two PBMC cultures are shown. Matched samples were collected on days 4–8 for the m-CSF culture (n = 8) and days 4–6 for the GM-CSF/IL4 cultures (n = 5). The editing rate was obtained from adherent cells in 10% FCS and m-CSF treated (macrophage-differentiation) wells and from non-adherent cells in supernatant in GM-CSF/IL4-treated (dendritic cell-differentiation) wells. (E) Sanger sequencing confirms C136U RNA editing in all four samples that showed high editing rates by RT-qPCR and high-throughput sequencing. Chromatograms show examples of a sample with a low editing rate (sample 1, day 3 in Table 1) and one with a high editing rate (sample 4, day 7 in Table 1). C136U variant is shown by arrows. [file peerj-01-152-s001.jpg]
