# Supplementary material for: Hypoxia-inducible C-to-U coding RNA editing downregulates SDHB in monocytes
Source: PeerJ. 2013 Sep 10;1:e152. doi: 10.7717/peerj.152 (PMC3775634; doi:10.7717/peerj.152)
Supplement: Table S1 [file peerj-01-152-s002.docx]

**Supplemental Table S1**

**Oligonucleotide primers and probe sequences**

| Forward (5’ to 3’) | Reverse (5’ to 3’) | Gene Target | Used in |
| --- | --- | --- | --- |
| TTGCCGGCCACAACCCTT | AGCCTTGTCTGGGTCCCATC | *SDHB* total transcripts  (exons 1-3) | AS qPCR, qPCR |
| TTGCCGGCCACAACCCTT | AGCCTTGTCTGG GTCCCATCA | *SDHB* C136U edited transcripts  (exons 1-3) | AS qPCR |
| FAM/AGACAGCTGCAGCCACAGCT |  | *SDHB* fluorescent probe  (exon 2) | qPCR probe |
| GGTCCTCAGTGGATGTAGGC | ACCAAGATCTTTAAAGGAACTCA | *SDHB* complete coding cDNA | High-throughput and Sanger sequencing |
| GTGGGAATTGTCGCCTAAGTG | ACATTTTCACATTAGAGATTCCCATT | *SDHD* complete coding cDNA | High-throughput sequencing |
| CCAGCAAAATGGAATTATCTTGT | CTCTCCTTCAATAGCTGGCTT | *SDHB* exon 2 genomic  (intronic primers) | Test of genomic DNA for C136T mutation |
| CTCGCTCCGTGGCCTTAGCT | TGGATGAAACCCAGACACATAGCA | B2-microglobulin | qPCR |
| CTCACCCAGGAGGGGAGAATC | CGATAGCAATTGCCCTGAAATCC | Cytidine deaminase | qPCR |
