# Supplementary material for: Hypoxia-inducible C-to-U coding RNA editing downregulates SDHB in monocytes
Source: PeerJ. 2013 Sep 10;1:e152. doi: 10.7717/peerj.152 (PMC3775634; doi:10.7717/peerj.152)
Supplement: Table S5 [file peerj-01-152-s006.docx]

**Supplemental Table S5**

**Transcript sequence variation at position 136 of *SDHB* mRNA in BodyMap study samples**

|  |  | *No. of reads with base call:* | | | | |  |  |  |  |
| --- | --- | --- | --- | --- | --- | --- | --- | --- | --- | --- |
| *Tissue* | *Source* | *A* | *C* | *G* | *T* | *Ambiguous* | *Inferred SDHB mRNA base* | *Total reads with non-ambiguous base* | *% reads with any base change* | *% reads indicating C>T editing* |
| Ovary | 47 y Af. American F | 2 | 0 | 200 | 0 | 0 | C | 202 | 0.99 | 0.99 |
| Breast | 29 y Caucasian F | 1 | 0 | 291 | 1 | 2 | C | 293 | 0.68 | 0.34 |
| Testis | 19 y Caucasian M | 1 | 1 | 291 | 1 | 0 | C | 294 | 1.02 | 0.34 |
| Kidney | 60 y Caucasian M | 3 | 1 | 328 | 0 | 0 | C | 332 | 1.2 | 0.90 |
| Thyroid | 60 y Caucasian F | 2 | 0 | 337 | 0 | 0 | C | 339 | 0.59 | 0.59 |
| White blood cells | 58 y Caucasian M | 6 | 0 | 346 | 0 | 0 | C | 352 | 1.7 | 1.70 |
| Heart | 77 y Caucasian M | 6 | 1 | 1249 | 1 | 1 | C | 1257 | 0.64 | 0.48 |
| Adipose tissue | 73 y Caucasian F | 0 | 0 | 146 | 0 | 0 | C | 146 | 0 | 0 |
| Adrenal gland | 60 y Caucasian M | 0 | 1 | 200 | 0 | 0 | C | 201 | 0.5 | 0 |
| Brain | 77 y Caucasian F | 0 | 1 | 179 | 0 | 0 | C | 180 | 0.56 | 0 |
| Colon | 68 y Caucasian F | 0 | 0 | 133 | 0 | 0 | C | 133 | 0 | 0 |
| Liver | 37 y Caucasian M | 0 | 2 | 573 | 0 | 1 | C | 575 | 0.35 | 0 |
| Lung | 65 y Caucasian M | 0 | 0 | 291 | 0 | 0 | C | 291 | 0 | 0 |
| Lymph node | 86 y Caucasian F | 0 | 0 | 158 | 0 | 0 | C | 158 | 0 | 0 |
| Prostate | 73 y Caucasian M | 0 | 2 | 284 | 0 | 0 | C | 286 | 0.7 | 0 |
| Skeletal muscle | 77 y Caucasian M | 0 | 0 | 553 | 0 | 1 | C | 553 | 0 | 0 |
