# Supplementary material for: Hypoxia-inducible C-to-U coding RNA editing downregulates SDHB in monocytes
Source: PeerJ. 2013 Sep 10;1:e152. doi: 10.7717/peerj.152 (PMC3775634; doi:10.7717/peerj.152)
Supplement: Table S6 [file peerj-01-152-s007.docx]

**Supplemental Table S6**

**Transcript sequence variation at position 136 of *SDHB* mRNA in ENCODE study samples**

|  |  |  | *Reads with base call:* | | | | |  |  |  |  |
| --- | --- | --- | --- | --- | --- | --- | --- | --- | --- | --- | --- |
| *Cell* | *Nature* | *Repli-cate* | *A* | *C* | *G* | *T* | *Ambiguous* | *Inferred SDHB mRNA base* | *Total reads with non-ambiguous base* | *% reads with any base change* | *% reads indicating C>T editing* |
| A549 | lung adenocarcinoma, 58 y Caucasian M | 1 | 1 | 2 | 690 | 1 | 1 | C | 694 | 0.58 | 0.14 |
| A549 | lung adenocarcinoma, 58 y Caucasian M | 2 | 1 | 0 | 951 | 0 | 0 | C | 952 | 0.11 | 0.11 |
| AG04450 | fetal lung fibroblast | 1 | 1 | 0 | 652 | 1 | 2 | C | 654 | 0.31 | 0.15 |
| AG04450 | fetal lung fibroblast | 2 | 0 | 0 | 593 | 1 | 0 | C | 594 | 0.17 | 0.00 |
| BJ | normal foreskin fibroblast, newborn M | 1 | 0 | 0 | 861 | 0 | 1 | C | 861 | 0.00 | 0.00 |
| BJ | normal foreskin fibroblast, newborn M | 2 | 0 | 0 | 600 | 0 | 2 | C | 600 | 0.00 | 0.00 |
| CD14+ monocytes | primary CD14+ cells from human leukapheresis production, from one donor | 1 | 8 | 0 | 413 | 0 | 3 | C | 421 | 1.90 | 1.90 |
| CD14+ monocytes | primary CD14+ cells from human leukapheresis production, from one donor | 2 | 9 | 0 | 342 | 0 | 0 | C | 351 | 2.56 | 2.56 |
| CD20+ B | primary B cells from two donors | 1 | 0 | 0 | 133 | 0 | 1 | C | 133 | 0.00 | 0.00 |
| CD20+ B | primary B cells from two donors | 2 | 0 | 0 | 256 | 0 | 5 | C | 256 | 0.00 | 0.00 |
| GM12878 | EBV-transformed peripheral blood B cell, Caucasian F | 1 | 3 | 0 | 734 | 1 | 1 | C | 738 | 0.54 | 0.41 |
| GM12878 | EBV-transformed peripheral blood B cell, Caucasian F | 2 | 0 | 3 | 1368 | 0 | 1 | C | 1371 | 0.22 | 0.00 |
| GM12891 | EBV-transformed peripheral blood B cell, Caucasian M | 1 | 0 | 0 | 256 | 2 | 0 | C | 258 | 0.78 | 0.00 |
| GM12891 | EBV-transformed peripheral blood B cell, Caucasian M | 2 | 1 | 0 | 128 | 0 | 0 | C | 129 | 0.78 | 0.78 |
| GM12892 | EBV-transformed peripheral blood B cell, Caucasian F | 1 | 0 | 0 | 151 | 0 | 0 | C | 151 | 0.00 | 0.00 |
| GM12892 | EBV-transformed peripheral blood B cell, Caucasian F | 2 | 0 | 1 | 159 | 0 | 0 | C | 160 | 0.63 | 0.00 |
| H1-ESC | embryonic stem cell | 1 | 4 | 0 | 591 | 0 | 1 | C | 595 | 0.67 | 0.67 |
| H1-ESC | embryonic stem cell | 2 | 0 | 0 | 495 | 1 | 3 | C | 496 | 0.20 | 0.00 |
| HCNM2 | TERT/CDK4-immortalized skeletal myoblasts, 41 y caucasian M | 1 | 0 | 1 | 719 | 0 | 0 | C | 720 | 0.14 | 0.00 |
| HCNM2 | TERT/CDK4-immortalized skeletal myoblasts, 41 y caucasian M | 2 | 0 | 0 | 524 | 0 | 0 | C | 524 | 0.00 | 0.00 |
| HCT-116 | colon carcinoma, M | 1 | 0 | 0 | 585 | 1 | 0 | C | 586 | 0.17 | 0.00 |
| HCT-116 | colon carcinoma, M | 2 | 0 | 0 | 569 | 0 | 0 | C | 569 | 0.00 | 0.00 |
| HeLa-S3 | cervical adenocarcinoma, 31 y Af.-American F | 1 | 9 | 2 | 762 | 2 | 3 | C | 775 | 1.68 | 1.16 |
| HeLa-S3 | cervical adenocarcinoma, 31 y Af.-American F | 2 | 0 | 0 | 1089 | 1 | 1 | C | 1090 | 0.09 | 0.00 |
| HepG2 | hepatocellular carcinoma, 15 y Caucasian M | 1 | 1 | 0 | 656 | 1 | 4 | C | 658 | 0.30 | 0.15 |
| HepG2 | hepatocellular carcinoma, 15 y Caucasian M | 2 | 0 | 0 | 485 | 0 | 0 | C | 485 | 0.00 | 0.00 |
| HMEC | mammary gland epithelial cells | 1 | 1 | 1 | 1321 | 1 | 4 | C | 1324 | 0.23 | 0.08 |
| HSMM | skeletal muscle myoblasts | 1 | 1 | 1 | 1125 | 1 | 2 | C | 1128 | 0.27 | 0.09 |
| HSMM | skeletal muscle myoblasts | 2 | 0 | 0 | 1124 | 0 | 0 | C | 1124 | 0.00 | 0.00 |
| HUVEC | primary umbilical vein endothelial cells | 1 | 0 | 0 | 431 | 0 | 3 | C | 431 | 0.00 | 0.00 |
| HUVEC | primary umbilical vein endothelial cells | 2 | 0 | 0 | 505 | 0 | 1 | C | 505 | 0.00 | 0.00 |
| IMR90 | lung fibroblast, 16 w gestation Caucasian F | 1 | 4 | 0 | 959 | 2 | 3 | C | 965 | 0.62 | 0.41 |
| IMR90 | lung fibroblast, 16 w gestation Caucasian F | 2 | 2 | 0 | 709 | 0 | 0 | C | 711 | 0.28 | 0.28 |
| K562 | CML leukemia pleural effusion, 53 y Caucasian F | 1 | 1 | 0 | 1520 | 1 | 1 | C | 1522 | 0.13 | 0.07 |
| K562 | CML leukemia pleural effusion, 53 y Caucasian F | 2 | 2 | 1 | 1504 | 0 | 5 | C | 1507 | 0.20 | 0.13 |
| MCF-7 | breast adenocarcinoma pleural effusion, 69 y Caucasian F | 1 | 2 | 0 | 742 | 2 | 4 | C | 746 | 0.54 | 0.27 |
| MCF-7 | breast adenocarcinoma pleural effusion, 69 y Caucasian F | 2 | 0 | 0 | 766 | 0 | 0 | C | 766 | 0.00 | 0.00 |
| NHEK | primary epidermal keratinocytes | 1 | 0 | 1 | 1120 | 1 | 1 | C | 1122 | 0.18 | 0.00 |
| NHEK | primary epidermal keratinocytes | 2 | 4 | 0 | 1247 | 1 | 0 | C | 1252 | 0.40 | 0.32 |
| NHLF | primary lung fibroblasts | 1 | 1 | 1 | 958 | 0 | 0 | C | 960 | 0.21 | 0.10 |
| NHLF | primary lung fibroblasts | 2 | 1 | 0 | 712 | 0 | 0 | C | 713 | 0.14 | 0.14 |
| SK-N-SH | brain neuroblastoma bone marrow metastasis, 4 y Caucasian F | 1 | 0 | 0 | 1073 | 0 | 2 | C | 1073 | 0.00 | 0.00 |
| SK-N-SH | brain neuroblastoma bone marrow metastasis, 4 y Caucasian F | 2 | 0 | 0 | 702 | 0 | 0 | C | 702 | 0.00 | 0.00 |
| SK-N-SH_RA | retinoic acid-treated brain neuroblastoma bone marrow metastasis, 4 y Caucasian F | 1 | 0 | 0 | 441 | 1 | 2 | C | 442 | 0.23 | 0.00 |
| SK-N-SH_RA | retinoic acid-treated brain neuroblastoma bone marrow metastasis, 4 y Caucasian F | 2 | 0 | 0 | 593 | 0 | 3 | C | 593 | 0.00 | 0.00 |
